# Diagnostic performance and utility of very high-resolution ultrasonography in diagnosing giant cell arteritis of the temporal artery

**DOI:** 10.1093/rap/rkz018

**Published:** 2019-07-05

**Authors:** Johnny K M Sundholm, Tom Pettersson, Anders Paetau, Anders Albäck, Taisto Sarkola

**Affiliations:** 1 Children's Hospital, University of Helsinki and Helsinki University Hospital, Helsinki, Finland; 2 Department of General Internal Medicine and Geriatrics, University of Helsinki and Helsinki University Hospital, Helsinki, Finland; 3 HUSLAB Meipa1 Pathology Laboratory, University of Helsinki and Helsinki University Hospital, Helsinki, Finland; 4 Haartman Institute, University of Helsinki, Helsinki, Finland; 5 Department of Vascular Surgery, University of Helsinki and Helsinki University Hospital, Helsinki, Finland

**Keywords:** giant-cell arteritis, diagnostics, ultrasound, ultrasound biomicroscopy, very-high resolution ultrasound

## Abstract

**Objective:**

Very-high resolution US (VHRU; 55 MHz) provides improved resolution and could provide non-invasive diagnostic information in GCA of the temporal artery. The objective of this study was to assess the diagnostic utility of VHRU-derived intima thickness (VHRU-IT) in comparison to high-resolution US halo-to-Doppler ratio (HRU-HDR) in patients referred for temporal artery biopsy.

**Methods:**

VHRU and HRU of the temporal artery were performed before a biopsy procedure in 78 prospectively recruited consecutive patients who had received glucocorticoid treatment for a median of 8 days (interquartile range 0–13 days) before imaging. Based on the final diagnosis and biopsy findings, the study population was divided into the following four groups: non GCA (*n* = 40); clinical GCA with no inflammation on biopsy (*n* = 15); clinical GCA with inflammation limited to adventitia (*n* = 9); and clinical GCA with transmural inflammation (TMI; *n* = 11).

**Results:**

Both VHRU and HRU were useful for identifying subjects with TMI, with VHRU outperforming HRU (area under curve: VHRU-IT 0.99, 95% CI 0.97, 1.00; HRU-HDR 0.74, 95% CI 0.52, 0.96; *P*=0.026). The diagnostic utility for diagnosing clinical GCA (negative biopsy) or inflammation limited to the adventitia was poor for both VHRU and HRU-HDR. From 5 days after initiation of glucocorticoid treatment, VHRU-IT was increased in eight of nine patients, whereas HRU-HDR was positive in three of seven patients. Both methods showed excellent inter-observer agreement (Cohen’s κ: VHRU-IT 0.873; HRU-HDR 0.811).

**Conclusion:**

In suspected GCA, VHRU allows non-invasive real-time imaging of TMI manifestations of the temporal artery wall. VHRU-derived intimal thickness measurement seems to be more sensitive than the halo sign and HRU-HDR in detecting TMI in patients with prolonged glucocorticoid treatment.

**Key messages**

- Very high-resolution US is useful for diagnosing GCA.
- Very high-resolution US can quantify temporal artery intima thickness non-invasively in GCA.
- Temporal artery intima thickness >0.3 mm predicts GCA with transmural inflammation.
- Both very high-resolution US and halo sign lack diagnostic utility in patients with limited inflammation on histology.

## Introduction

GCA is the most common inflammatory vasculopathy in adults [1]. It affects predominantly medium and large arteries and has a global incidence of 10/100 000, with a higher incidence in northern Europe [2, 3]. The peak incidence is between the ages of 70 and 80 years, and the disease rarely occurs in individuals younger than 50 years. It is more common in women, and there is a 50% co-morbidity with polymyalgia rheumatica [4].

In 1997, Schmidt *et al.* [5] described a hypodense perivascular halo sign on high-resolution US (HRU) consistent with perivascular oedema in GCA. Further US-derived parameters, including arterial occlusion, lack diagnostic utility [5]. Operator dependence impacting on the subjective interpretation of the US image requires optimization and standardization of imaging protocols in the diagnostic process [6]. Cut-off values for diagnosis have been reported to reduce the rate of false positives [7–10].

The time line in the response to glucocorticoid treatment is another challenge, with diagnostic radiological features reverting in up to 50% of subjects after 2–4 days [11]. Meta-analyses report a 68–77% sensitivity and an 83–96% specificity for the unilateral halo sign and a 43% sensitivity and 100% specificity for a bilateral halo sign [12–15]. In recent reviews, the halo sign has been described as a fairly reliable sign of GCA, and it has been proposed for inclusion in the diagnostic criteria in the assessment of suspected GCA [16–19].

Non-invasive very-high resolution US (VHRU; 55 MHz, axial resolution 0.04 mm) allows imaging of superficial structures of the nearfield in more detail compared with conventional US frequencies (<20 MHz), and we have recently validated non-invasive measurements of the arterial intima using VHRU [20, 21]. Previous small sample case series of temporal artery VHRU imaging in the setting of GCA have shown promising results, but no comparison with conventional HRU imaging has been made [22–24]. There are no previous studies on the diagnostic utility of VHRU-derived intima thickness in the setting of GCA.

The aim of this study was to assess the following: the utility of VHRU in measuring vascular wall layer dimensions in patients with suspected GCA; and the diagnostic utility of the VHRU-derived vascular wall layer dimensions compared with conventional HRU in suspected GCA of the temporal artery. Our hypothesis was that the increased resolution of VHRU would allow a more detailed and specific evaluation of the arterial wall than HRU and that this could be of benefit in the diagnostic process of suspected temporal artery GCA.

## Methods

We prospectively recruited 78 consecutive patients between August 2015 and May 2018 with clinically suspected GCA referred to the unit of vascular surgery for biopsy of the temporal artery as part of routine GCA diagnostics. Two patients declined to participate, and histological information was missing in three patients. The remaining 75 patients were included in the final analysis. Subject characteristics and symptoms were recorded at presentation using a standard questionnaire, filled in by the investigator (J.K.M.S.), assessing smoking history, the presence of symptoms, symptom duration and findings on clinical examination, such as temporal tenderness, prominent temporal artery and diminished temporal artery pulsation. Patient records were reviewed to assess laboratory findings, including biopsy results, background information on treatments and medications, initiation date of CS treatment and final diagnosis. The ethics board of the Helsinki University Hospital approved the research protocol, and written consent was obtained from all participants at enrolment.

### US assessment

Vascular US of the temporal artery was performed 1 h before biopsy with HRU followed by VHRU by one skilled investigator (J.K.M.S.). No HRU images were obtained for the initial 22 subjects, and HRU scanning was performed in 56 of 78 patients overall. VHRU scanning was performed in all 78 subjects in accordance with the study protocol.

High-resolution US imaging was performed using a L10-22-RS linear vascular transducer (frequency range 10–22 MHz; GE LOGIQ e, model year 2015, GE Healthcare, Chicago, IL, USA) for the first 20 subjects and an UHF 22 (frequency range 10–22 MHz, axial resolution 0.110 mm, corresponding to three image pixels) linear transducer (Vevo MD, model year 2017, VisualSonics, Toronto, ON, Canada) for the subsequent subjects.

Very high-resolution US scanning was performed using an RMV 708 (frequency range 22–83 MHz, 0.045 mm, three pixels) mechanical transducer (Vevo 770, model year 2005; VisualSonics) system for the first 41 subjects. Owing to breakdown of the equipment beyond repair, the remaining subjects were scanned using a UHF70 (frequency range 29–71 MHz, 0.036 mm, three pixels) transducer (Vevo MD, model year 2017, VisualSonics).

The superficial temporal artery were screened bilaterally with HRU and VHRU, both transversely and longitudinally, from the zygomatic arch to the bifurcation and further, including both frontal and parietal branches. Images were stored as moving digital cine clips. The scanning area was marked on the skin at the end of imaging to guide the surgeon and to ensure matching of biopsy and imaging locations.

High-resolution US images and VHRU images were coded with a study number and stored as moving cine-clips and evaluated offline on different occasions at ≥2 weeks apart by the investigator (J.K.M.S.). The HRU images were evaluated for the halo sign, consisting of a visible dark halo around the lumen of the artery, and by measuring the ratio of the halo to colour Doppler areas [halo-to-Doppler ratio (HDR)], with area borders traced manually. The assessment was made offline at end-diastole using ImageJ v.1.51J8 (US National Institutes of Health, Bethesda, MD, USA) [25] for GE images and Vevo Lab v.2.0.0 for Vevo MD images.

The VHRU images were assessed by the investigator (J.K.M.S.), and far wall arterial wall layer thickness was quantified. Intima thickness (IT) and intima–media thickness were measured both as a mean of three measurements for comparison with histology and as a maximal value that was used for diagnostic purposes. All measurements were made at end-diastole from the images offline with vendor software Vevo v.3.0.0 (Vevo 770) and VevoLab v.2.0.0 (Vevo MD) using the far wall leading-to-leading edge method with electronic callipers before histological processing.

All HRU cine clips were evaluated independently for halo sign and HRU-HDR, followed by evaluation of all VHRU cine clips for maximal VHRU-derived intima thickness (VHRU-IT) by a second skilled investigator (T.S.) blinded to patient characteristics and histological diagnosis to assess diagnostic utility.

### Temporal artery biopsy

After US imaging, the biopsy procedure was performed as routine GCA diagnostics at the Department of Vascular Surgery at Helsinki University Hospital. In the setting of suspected vasculitis on US, the biopsy was taken from that area. In the setting of a normal US appearance, the biopsy was taken from the more symptomatic side.

The biopsy was fixed in formalin, cut into transverse sections (multiple layers) and stained with Haematoxylin and Eosin and Verhoff’s elastic stain [26]. A subset of histologically uncertain cases was evaluated further using a T-lymphocyte immunohistochemical CD3^+^ stain [27]. Biopsies were evaluated for vascular pathology at a certified pathology unit (HUSLAB).

Histological diagnosis was graded as follows: 1, no inflammation; 2, perivascular inflammation limited to the adventitia (ILA); and 3, transmural inflammation (TMI) affecting the intimal and medial layers of the vascular wall [28].

Histological assessments were evaluated using optic microscopy, photographed at ×10 zoom, and vascular dimensions were measured offline by the investigator (J.K.M.S.) using ImageJ v.1.51J8 with electronic callipers, as a mean of 10 measurements, with IT, IMT and thickness of the adventitia measured separately.

### Diagnosis and study groups

Patient records were screened for a period of 6 months after biopsy. The final diagnosis was set by rheumatologists at the Helsinki University Hospital Department of Rheumatology based on symptoms, clinical signs, laboratory findings, biopsy, evidence of large vessel vasculitis on PET CT or MRI, treatment response and, in the case of an uncertain diagnosis, a careful differential diagnostic work-up during follow-up. To assess whether US findings were related to histological findings, we divided GCA-positive patients further into three groups: GCA with no inflammation; GCA with ILA; and GCA with TMI.

### Data analysis

Results are presented as the mean (s.d.), median (range) and proportions as appropriate. Variables were tested for normal distribution using the Shapiro–Wilk test, and groups were compared using ANOVA for normally distributed variables (post-hoc Bonferroni test). The Kruskal–Wallis test was used for continuous variables not conforming to normality (post-hoc Dunn–Bonferroni test) and the Fisher–Freeman–Halton test for categorial variables (post-hoc independent Fisher comparisons with Bonferroni-adjusted levels of significance). Inter-observer variability for HRU-HDR and VHRU IT was assessed using the intraclass correlation coefficient.

The diagnostic performance of HRU-HDR and VHRU-IT was assessed using receiver operating characteristic curves based on assessments performed independently by a second blinded investigator (T.S.) comparing individual histological groups separately in addition to a clustered group of biopsy-positive patients using the non-GCA group as a negative reference. HRU-HDR and VHRU-IT methods were compared using a paired test of equality for the area under the curve [29]. Cut-off values for different parameters were evaluated using sensitivity–specificity charts optimizing the positive likelihood ratio (LHR+). Statistical analysis was performed using SPSS v.24 (IBM, Armonk, NY, USA) and StataMP v.15.1 (Stata Corp., Houston, TX, USA).

## Results

**Fig. 1:**
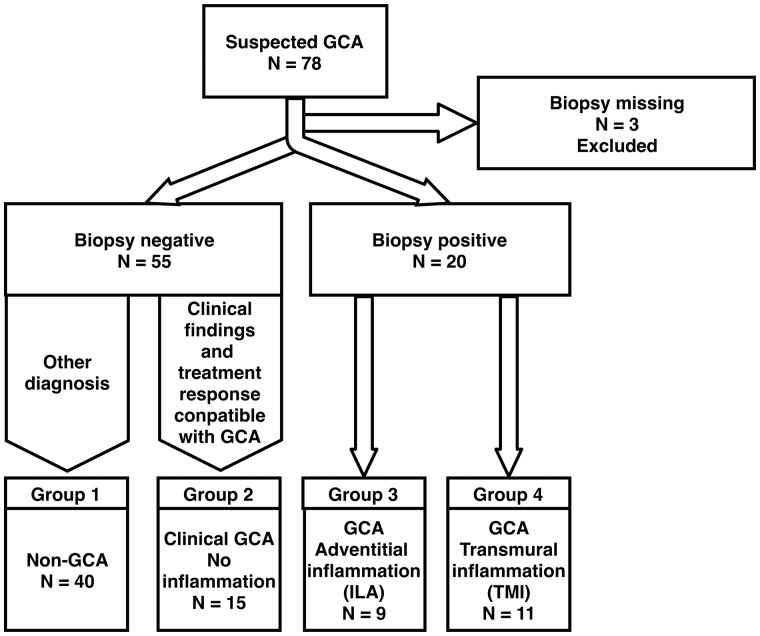
Flowchart of patient recruitment Patient recruitment and allocation into groups according to clinical criteria and histology in biopsy.

**Fig. 2:**
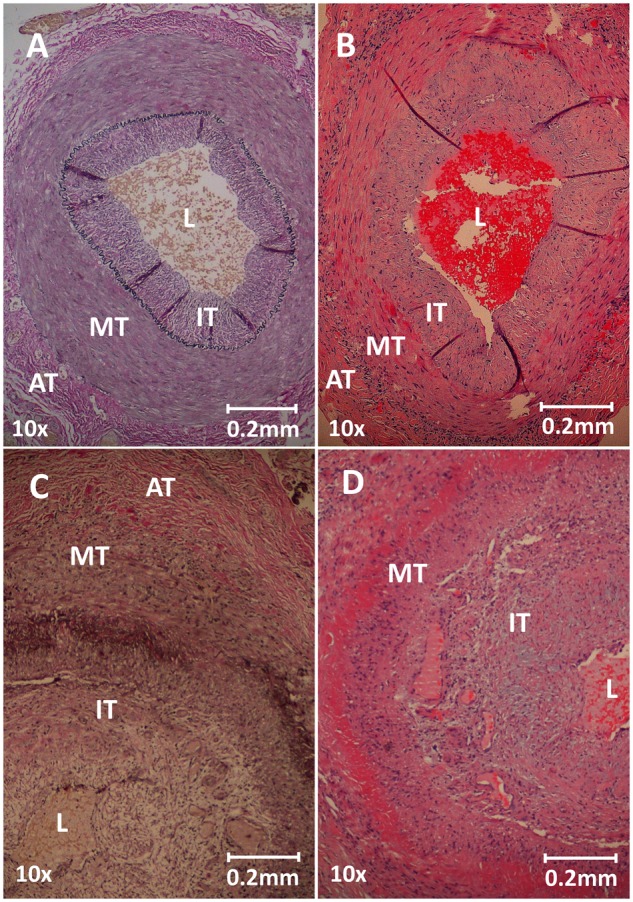
Histological grading of inflammation (**A**) Histology of Verhoff’s elastic stain of a temporal artery without inflammation (no GCA group and clinical GCA without inflammation group). (**B**) Histology of Haematoxylin and Eosin stain of a temporal artery with minor ILA. Note the streak of inflammatory cells throughout the media–adventitia border. (**C**, **D**) Verhoff’s elastic stain (**C**) and Haematoxylin and Eosin stain (**D**) of a temporal artery with TMI. AT: adventitia; ILA: inflammation limited to the adventitia; IT: intima; L: lumen; MT: media; TMI: transmural inflammation.

Seventy-eight consecutive patients were recruited prospectively for this study, and imaging was feasible in all subjects. The subjects were allocated into four groups according to the final clinical and histological diagnosis as follows (Figs 1 and 2). In group 1, the patients with no GCA, who had no inflammation on histology and a GCA diagnosis considered unlikely by a rheumatologist (*n* = 42), the final diagnoses were polymyalgia rheumatica (*n* = 13), fever of unknown origin (*n* = 6), non-arteritic anterior ischaemic optic neuropathy (*n* = 6), bacterial infection (*n* = 5), neurological diseases (migraine, stroke, haemorrhage, transient ischaemic attack, *n* = 6), malignancy (*n* = 3) and other rheumatic diseases (SS, RA and granulomatosis with polyangiitis, *n* = 3). Group 2 patients were diagnosed with GCA, without inflammation on histology but diagnosed on clinical grounds by a rheumatologist (*n* = 16). The third group of patients had GCA, with minor inflammation on histology limited to the perivascular structures and adventitia (ILA, *n* = 9). The fourth group of patients were diagnosed with GCA, with transmural inflammation on histology (TMI, *n* = 11).

The biopsy procedure failed in three subjects, and histological information was thus missing in one clinical GCA and two non-clinical GCA subjects. These subjects were excluded from the final analysis. Six biopsy specimens (three from group 1 and two from group 2) were sectioned diagonally, precluding reliable measurements of vascular dimensions, although not limiting diagnostics.

Three biopsy samples with very limited inflammation confined to the perivascular area and/or vasa vasorum were allocated within the ‘no inflammation’ subgroups owing to the uncertain significance of this finding [30]. CS treatment had been initiated in 56 patients (72%) before recruitment, and 50 patients (64%) had received CSs for ≥5 days.

The main patient characteristics are presented in Table 1, with further data in Supplementary Table 1, available at *Rheumatology Advances in Practice* online. Age was significantly higher, temporal arteries more prominent and the number of ACR classification criteria higher in the TMI group than in the other groups. There were no significant differences in patient characteristics, laboratory results or findings on clinical examination when comparing the ILA group and biopsy-negative GCA *vs* non-GCA. There was a significant difference in the presence of aortitis on PET/MRI in the group comparison overall, but the results were statistically non-significant in further between-group post-hoc comparisons.

**Table 1:** Subject characteristics by study group

Study group	Non-GCA	GCA, biopsy negative	GCA, ILA	GCA, TMI	*P*-value
*n*	40	15	9	11	
Age, mean (s.d.), years	66.2 (10.7)	62.2 (8.5)	66.4 (10.7)	77.0 (5.6)^a^	0.005
Sex (female), *n* (%)	23 (58)	10 (67)	5 (55)	6 (55)	0.872
CS treatment before biopsy, *n* (%)	25 (63)	14 (93)	7 (78)	9 (82)	0.114
CS duration before biopsy, median (range), days	7 (0–157)	12 (0–71)	9 (0–65)	6 (0–37)	0.591
Prominent temporal arteries on inspection, *n* (%)	5 (13)	3 (20)	2 (22)	8 (73) ^a^	0.001
Polymyalgia rheumatica, *n* (%)	15 (38)	10 (67)	4 (44)	4 (36)	0.592
Aortitis, *n* (%)	1 (3)	3 (20)	2 (22)	1 (9)	0.043
ACR classification criteria					
Criteria present excluding biopsy, median (range), *n*/4	2.5 (0–4)	3 (2–4)	2 (2–4)	4 (1–4) ^a^	0.008
Age >50 years, *n* (%)	39 (95)	15 (100)	8 (89)	11 (100)	0.521
Headache, *n* (%)	23 (58)	13 (87)^a^	4 (44)	9 (82)	0.012
Scalp tenderness/decreased temporal artery pulsation on palpation, *n* (%)	17 (43)	9 (60)	3 (33)	9 (82)	0.068
ESR (>50 mm/h), *n* (%)	21 (53)	10 (67)	8 (89)	9 (82)	0.141
Laboratory tests before treatment					
CRP, median (range), mg/l	63 (<3–372)	93 (9–211)	139 (19–321)	95 (17–233)	0.210
ESR, median (range), mm/h	57 (5–129)	66 (9–128)	84 (29–127)	79 (12–114)	0.183

Results are presented as the mean (s.d.), median (range) or *n* (%). *P*-values represent results for group comparisons with ANOVA (post-hoc: Bonferroni test), Fisher–Freeman–Halton exact test (post-hoc: independent Fisher’s exact test with Bonferroni-adjusted significance levels) or Kruskal–Wallis test (post-hoc: Dunn–Bonferroni test).

a Differs significantly from non-GCA group in post-hoc analysis, at *P *<0.05.

ILA: inflammation limited to adventitia; TMI: transmural inflammation.

### Very high-resolution US and vascular dimensions

**Fig. 3:**
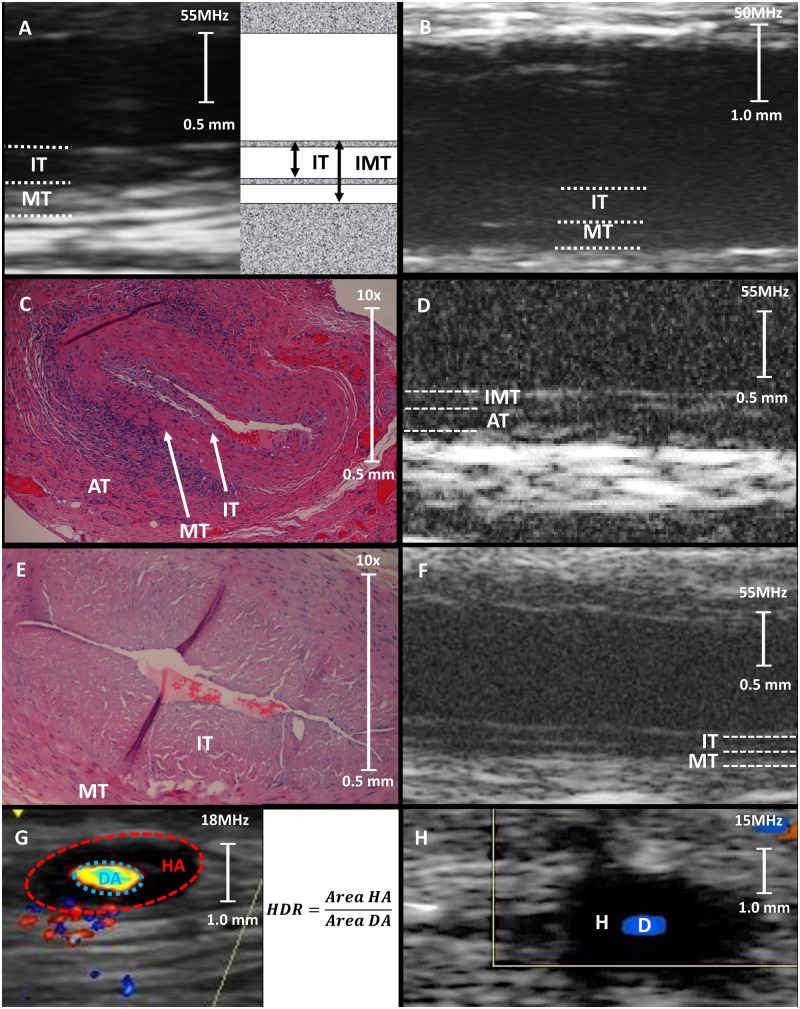
Examples of high-resolution US, very high-resolution US and histology images (**A**) IT and IMT measurement with VHRU. (**B**) VHRU of temporal artery with TMI. (**C**, **D**) H&E-stained histological section (**C**) and VHRU (**D**) of temporal artery with TMI, a VHRU-IT false negative. (**E**, **F**) H&E-stained histological section (**E**) and VHRU (**F**) of a temporal artery in a non-GCA patient, with cardiovascular risk factors (IT 0.2 mm). (**G**, **H**) US images of the HDR measurement with HRU (**G**), and a positive halo sign in a patient with TMI (**H**). AT: adventitia thickness; D: Doppler; DA: Doppler area; H: halo; HA: halo area; HDR: halo-to-Doppler ratio; H&E: Haematoxylin and Eosin; IMT: intima–media thickness; IT: intima thickness; MT: media thickness; TMI: transmural inflammation; VHRU: very high-resolution US.

Very high-resolution US allowed for measurements not only of IMT but also of IT in subjects with intimal thickening. Intima thickness and IMT (Fig. 3A) agreed well with histological measurements (intraclass correlation coefficient 0.897 and 0.894, respectively).

Histological and US measurements of IT and IMT in the TMI group differed significantly from the other groups. No difference was seen, however, when comparing isolated media thickness, suggesting that thickening of the intima is the main contributor to increased vascular wall thickness in temporal arteries with TMI (Table 2). The adventitia was significantly thicker on histology in the TMI group compared with the other groups. However, we were unable to quantify the thickness of the adventitia reliably in VHRU images with the leading-to-leading edge measurement owing to histological thickness bordering or below the axial resolution of VHRU in a majority of the assessed arteries.

**Table 2:** High-resolution US halo, very high-resolution US-derived arterial wall layer thickness and histological arterial wall layer thickness

Study group	Non-GCA *n* = 40	GCA, Biopsy negative *n* = 15	GCA, ILA *n* = 9	GCA, TMI *n* = 11	*P*-value
HRU; *n*	31	9	7	9	
Halo, *n* (%)	5 (16)	1 (11)	2 (29)	5 (56)	0.083
Halo-to-Doppler ratio	1.4 (1.0–2.8)	1.4 (1.2–1.9)	1.4 (1.0–4.2)	2.4 (1.5–9.3)^a^	0.007
HDR >2.0, *n* (%)	2 (6)	0 (0)	2 (29)	5 (56)^a^	0.003
VHRU					
Measurable IT, *n*	31	9	9	11	
Mean IT, median (range), mm	0.12 (0.06–0.23)	0.09 (0.06–0.14)	0.14 (0.06–0.22)	0.28 (0.06–0.53)	<0.001
Maximal IT, median (range), mm	0.16 (0.06–0.28)	0.11 (0.08–0.28)	0.16 (0.08–0.25)	0.40 (0.06–0.70)^a^	<0.001
Maximal IT >0.30 mm, *n* (%)	0 (0)	0 (0)	0 (0)	10 (91)^a^	<0.001
IMT, *n*	40	15	9	11	
IMT, median (range), mm	0.22 (0.08–0.42)	0.22 (0.09–0.29)	0.23 (0.17–0.28)	0.55 (0.22–0.834)^a^	<0.001
Maximal IMT, median (range), mm	0.27 (0.09–0.49)	0.26 (0.10–0.43)	0.28 (0.18–0.55)	0.69 (0.31–1.10)	<0.001
Histology, *n*	37	12	9	11	
IT, median (range), mm	0.11 (0.01–0.24)	0.09 (0.02–0.18)	0.14 (0.08–0.19)	0.38 (0.04–0.90)^a^	<0.001
MT, median (range), mm	0.16 (0.05–0.36)	0.16 (0.08–0.25)	0.17 (0.11–0.34)	0.20 (0.11–0.41)	0.442
IMT, median (range), mm	0.28 (0.07–0.49)	0.24 (0.10–0.41)	0.31 (0.24–0.50)	0.59 (0.15–1.31)^a^	<0.001
AT, median (range), mm	0.06 (0.02–0.12)	0.06 (0.03–0.08)	0.07 (0.05–0.14)	0.14 (0.05–0.23)^a^	<0.001

Halo-to-Doppler ratio and vascular dimensions assessed by the primary investigator (J.K.M.S.). Results are presented as the median (range) or *n* (%). *P*-values represent results for group comparisons with the Fisher–Freeman–Halton exact test (post-hoc: independent Fisher’s exact test with Bonferroni-adjusted significance levels) and the Kruskal–Wallis test (post-hoc: Dunn–Bonferroni test). Note that IT was not measurable in VHRU imaged in subjects with IT<0.06 mm.

a Differs significantly from all other groups in post-hoc analysis at *P *<0.05.

AT: adventitia thickness; HDR: halo-to-Doppler ratio; HRU: high-resolution US; IMT: intima–media thickness; ILA: inflammation limited to adventitia; IT: intima thickness; MT: media thickness; TMI: transmural inflammation; VHRU: very high-resolution US.

Sensitivity–specificity charts were evaluated to determine optimal cut-off values for IT and IMT optimizing LHR+. Suggested diagnostic cut-off values were as follows: IT >0.3 mm (sensitivity 90.9%, 95% CI 58.7, 99.8%; specificity 100%, 95% CI 91.1, 100.0%; LHR+ not applicable; LHR− 0.1); IMT >0.44 mm (sensitivity 72.7%, 95% CI 39.0, 94.0%; specificity 100%, 95% CI 90.5, 100.0%; LHR+ not applicable; LHR− 0.3) in the common superficial temporal artery.

The vascular walls in TMI subjects who had not received glucocorticoids were evidently hypoechoic, and the borders of the vascular dimensions were challenging to distinguish (Fig. 3B). The VHRU-IT in the TMI group remained increased for up to 37 days after initiation of treatment (Supplementary Fig. 1B, available at *Rheumatology Advances in Practice* online).

Only one subject with TMI (male, 77 years of age) did not show intimal thickening on histology or VHRU (IT 0.03 mm), with evident thickening of the adventitia, making interpretation of the US image challenging (Fig. 3C, D). The IT exceeded 0.30 mm in all other patients in the TMI group.

There was a broad variation in IT within the non-GCA group, with 85% >0.1 mm and 19% >0.2 mm (healthy range 0.01–0.03 mm), supposedly related to advanced age and cardiovascular risk factors (Fig. 3E, F) [31, 32].

### Halo sign and halo-to-Doppler ratio

The prevalence of the halo sign in each group is presented in Table 2. There was no significant difference between groups. The halo sign had a sensitivity of 55.6% and a specificity of 83.9% to detect TMI (LHR+ 3.4; LR− 0.5).

Measures of HDR (Fig. 3G) in HRU images by group are presented in Table 2. The HDR was significantly higher in the TMI group than in the other groups, whereas no significant difference was seen in HDR between ILA and biopsy-negative groups compared with non-GCA patients. Sensitivity–specificity charts provided HDR >2.0 (representing a mean halo thickness of 0.23 mm) as an optimal cut-off to predict TMI with increased specificity compared with the halo sign (sensitivity 55.6%, 95% CI 21.2, 86.3%; specificity 93.5%, 95% CI 77.9, 99.1%; LHR+ 8.6; LHR− 0.5).

All four halo sign and HRU-HDR false negatives in the TMI group were in patients treated with CSs for >5 days (HRU-HDR mean 2.8, range 1.49–7.53), with HRU-HDR being higher in patients having a shorter treatment duration (HRU-HDR mean: 6.9, range 4.5–9.3) (Fig. 3H and Supplementary Fig. 1, available at *Rheumatology Advances in Practice* online).

### Diagnostic utility of halo-to-Doppler ratio and very high-resolution US

**Fig. 4:**
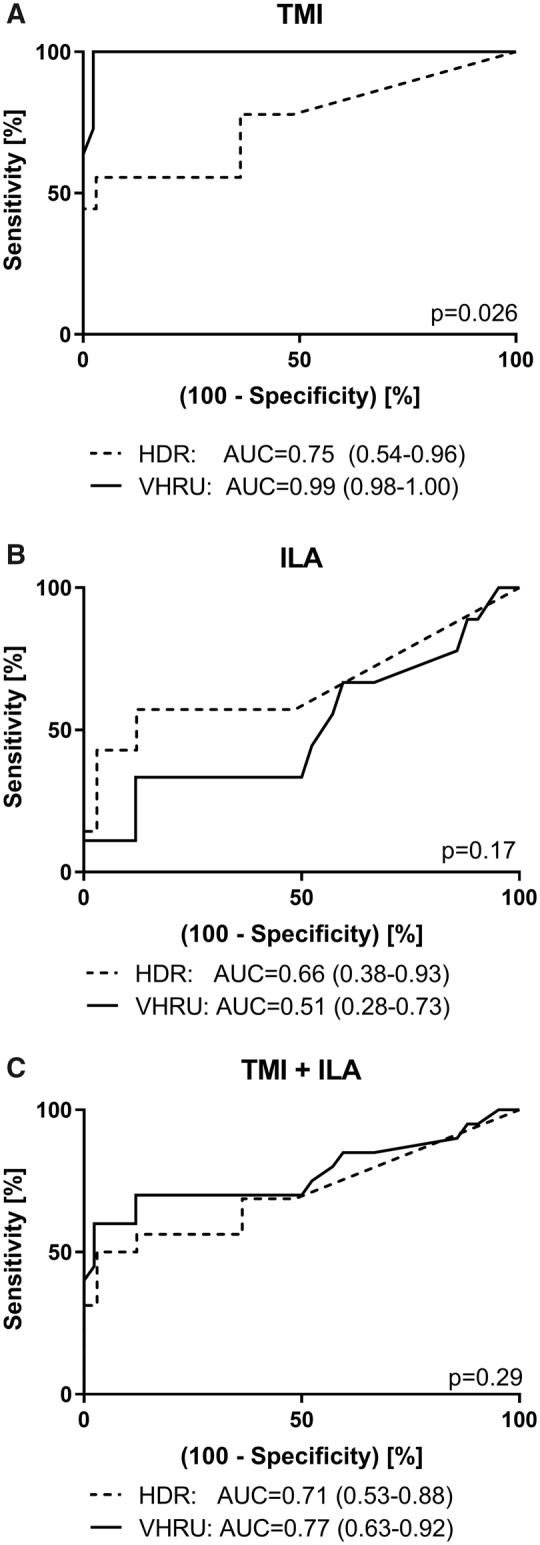
Receiver operating characteristic curves describing diagnostic utility of halo-to-Doppler ratio and very high-resolution US-derived intima thickness Receiver operating characteristic curves describing diagnostic utility of halo-to-Doppler ratio and VHRU-derived intima thickness. (**A**) TMI. (**B**) ILA. (**C**) ILA and TMI combined (i.e. inflammation on histology). AUC: area under curve; HDR: halo-to-Doppler ratio; ILA: inflammation limited to the adventitia; TMI: transmural inflammation; VHRU: very-high resolution US.

To evaluate the diagnostic utility of HRU-HDR and VHRU IT in GCA, the images were analysed by a second observer blinded to patient characteristics. Inter-observer agreement for HDR-HRU and VHRU-IT measurements was high (intraclass correlation coefficient 0.792 and 0.904, respectively). Inter-observer agreements for both IT >0.3 mm and HDR >2.0 cut-off values for diagnosis were also high (Cohen’s κ 0.873 and 0.811, respectively). Diagnostic utility for the different methods was evaluated further using receiver operating characteristic analyses (Fig. 4). Both methods were useful for identifying patients with TMI on histology, with VHRU-IT-derived measurements outperforming HRU-HDR (area under curve: VHRU-IT 0.99, 95% CI 0.98, 1.00; HRU-HDR 0.75, 95% CI 0.54, 0.96; *P *= 0.026). Neither method was able to identify patients with ILA (area under curve: VHRU-IT 0.51, 95% CI 0.28, 0.73; HRU-HDR 0.66, 95% CI 0.38, 0.93; for difference, *P *= 0.17). Suggested diagnostic cut-off values for the second operator (T.S.) were similar to those of the primary operator: HRU-HDR >2.1 (sensitivity 55.6%, 95% CI 21.2, 86.3%; specificity 96.7%, 95% CI 84.2, 99.9%; LHR+ 16.8; LHR− 0.5) and VHRU-IT >0.28 mm (sensitivity 100%, 95% CI 73.5, 100.0%; specificity 97.7%, 95% CI 87.4, 99.9%; LHR+ 43.5; LHR− not applicable).

## Discussion

The aims of the present study were to evaluate the diagnostic utility of VHRU for diagnosing GCA and to compare the diagnostic value of VHRU with that of HRU. The improved resolution of VHRU allows for measurements not only of IMT but also of IT. Intimal thickening is a key feature of GCA, and the present study shows that histological temporal artery IT can be quantified with VHRU. Increased IT is diagnostic for GCA in the setting of TMI but not in the setting of milder GCA forms of histological vascular wall inflammation limited to the adventitia (ILA). This is in line with previous studies using VHRU in patients with GCA, where both vascular wall thickening and perivascular halo predicted histological TMI on histology [23, 24]. Non-invasive VHRU-derived measurements of both IMT and IT were diagnostic for TMI, but the thickening of the vascular wall was predominantly related to intimal thickening, suggesting superiority of VHRU-IT. The high prevalence of non-inflammatory intimal thickening in the non-GCA group did not significantly impact on the diagnostic performance of VHRU-IT. Our diagnostic cut-off for vascular US-derived IMT values are in line with those previously published [10]. The thickness of the adventitia was significantly increased on histology in the TMI group compared with other groups. Adventitia VHRU measurements were, however, challenging and unreliable, limiting its utility as a diagnostic parameter.

In the present study, we implemented a simple objective measure to reduce subjectivity and to increase specificity, the HDR, in addition to the previously reported halo sign [9]. The diagnostic utility for HRU-HDR in patients with TMI on histology was in line with previous publications that have assessed the diagnostic utility of the halo sign [17]. Likewise, we found more false negatives in patients who had been under glucocorticoid treatment for >5 days, with an HDR greatly exceeding the cut-off value in TMI patients with shorter treatment duration [11]. This suggests that a higher HDR cut-off could potentially increase specificity in the pre-treatment setting.

Although the periluminal halo diminishes within days after initiation of glucocorticoid treatment, the intimal thickening was seen in patients with prolonged glucocorticoid treatment before imaging. This implies that measurement of VHRU-IT is more resilient to treatment [33, 34]. Nevertheless, we recognized that in vessels with considerable oedema the VHRU measurement of vascular wall layers was technically difficult owing to the distortion of the vascular wall and diminished US interphases.

The diagnostic utility of VHRU-IT seems to be limited to patients with TMI, with no added diagnostic value in patients with ILA or in those with no inflammation on histology and without intimal thickening. This limitation is not only limited to VHRU, because the sensitivity of the perivascular Doppler sign is also reduced in these patients [35]. This is not only related to imaging, because the predictive value of perivascular inflammation and ILA on histology has recently been criticized [30, 36]. Further limitations of VHRU-IT could potentially include a high prevalence of age-related intimal thickening, increasing the cut-off value for diagnosis [37]. In our study population, however, GCA-related intimal thickening exceeded age-related intimal thickening in almost all cases.

The limitations of this study include lack of follow-up VHRU data and the fact that in many cases imaging and biopsy was performed after several days of glucocorticoid treatment. This probably reduces the sensitivity of the established halo sign, because it diminishes the periluminal oedema, thus decreasing the diagnostic cut-off level and making the test less specific. Furthermore, HRU imaging was not performed in the initially recruited patients. This reduces comparability between methods and with previous studies and limits the utility of the results in a pre-treatment setting. Another limitation is the high number of ILA and biopsy-negative patients in our sample. This might be related to the prolonged glucocorticoid treatment, and a high proportion being referred to temporal artery biopsy owing to pyrexia of unknown origin in our tertiary care study setting. Our study setting, however, reflected clinical practice and allowed us to match US images with histology. We were able to verify that the US-derived dimensions, in fact, corresponded to the histological measures. Another limitation is that images were gathered from the temporal artery only, not including axillar arteries, as is currently recommended [19, 38]. An additional strength is that we gathered images with both the established HRU method and with VHRU, which enabled a direct comparison of the coded images with the investigator blinded to clinical characteristics and histological diagnosis.

### Conclusions

Very high-resolution US-derived measurement of temporal artery IT in patients with suspected GCA seems useful for non-invasive real-time diagnosis of TA manifestations of GCA. The diagnostic utility of the VHRU method does not seem to be limited by glucocorticoid treatment to the same extent as the established perivascular halo. VHRU provides a potential non-invasive method for both real-time diagnosis and longitudinal follow-up of the temporal artery wall in GCA patients in the clinic.

*Funding*: This work was supported by grants from the Sigrid Jusélius Foundation, the Medical Society of Finland, and Finnish Foundation for Pediatric Research, Perklén Foundation, Medicinska understödsföreningen Liv och Hälsa, and the Stockmann Foundation.

*Disclosure statement*: The authors have declared no conflicts of interest.

## Supplementary Material

rkz018_Supplementary_Data
