# Supplementary material for: Diagnostic performance and utility of very high-resolution ultrasonography in diagnosing giant cell arteritis of the temporal artery
Source: Rheumatol Adv Pract. 2019 Jul 5;3(2):rkz018. doi: 10.1093/rap/rkz018 (PMC6735832; doi:10.1093/rap/rkz018)
Supplement: rkz018_Supplementary_Data [file rkz018_supplementary_data.docx]

**Supplement for Sundholm et al. Diagnostic performance and utility of very-high resolution ultrasonography in diagnosing giant cell arteritis of the temporal artery.**

Supplemental Table 1. Subject characteristics by study group.

| Group | Non-GCA | GCA, Biopsy negative | GCA, Inflammation limited to adventitia (ILA) | GCA, Transmural inflammation (TMI) |
| --- | --- | --- | --- | --- |
| N | 42 | 16 | 9 | 11 |
| *Questionnaire* |  |  |  |  |
| Weight loss | 10 (28%) | 7 (47%) | 8 (89%)^a^ | 3 (27%) |
| Night sweats | 25 (63%) | 11 (73%) | 7 (78%) | 7 (64%) |
| Fatigue | 26 (65%) | 13 (87%) | 8 (89%) | 7 (64%) |
| Nausea | 8 (20%) | 2 (13%) | 2 (22%) | 1 (9%) |
| Visual changes | 14 (33%) | 6 (40%) | 2 (22%) | 5 (45%) |
| Stroke/TIA | 6 (15%) | 1 (7%) | 0 (0%) | 0 (0%) |
| Jaw claudication | 8 (20%) | 6 (40%) | 2 (22%) | 7 (64%) |
| Claudication of extremities | 15 (38%) | 5 (33%) | 4 (44%) | 4 (36%) |
| Cough, sore throat, hoarseness | 12 (30%) | 8 (53%) | 1 (11%) | 6 (45%) |
| Previous smoker | 21 (53%) | 7 (15%) | 7 (78%) | 4 (36%) |
| Active smokers | 20 (50%) | 6 (40%) | 6 (67%) | 4 (36%) |
| *Hospital records* |  |  |  |  |
| Hypertension | 20 (50%) | 5 (33%) | 3 (33%) | 8 (73%) |
| Diabetes | 9 (23%) | 4 (27%) | 1 (11%) | 4 (36%) |
| Hypercholesterolaemia | 15 (38%) | 3 (20%) | 2 (22%) | 4 (36%) |
| Coronary artery disease | 7 (18%) | 2 (13%) | 0 (0%) | 1 (9%) |
| Hypothyroidism | 4 (10%) | 1 (7%) | 0 (0%) | 3 (27%) |
| *Laboratory tests prior to glucocorticoid treatment* |  |  |  |  |
| Haemoglobin [g/l] | 129 (102-181) | 123 (105-148) | 126 (94-138) | 115 (101-133) |
| Anaemia^b^ | 23 (58%) | 9 (60%) | 7 (78%) | 10 (91%) |
| Leucocytes [E9/l] | 8.8 (4.7-17.9) | 10.4 (6.7-14.3) | 9.8 (4.6-11.6) | 9.1 (3.6-11.9) |
| Leucocytosis [>8.2E9/l] | 33 (80%) | 13 (93%) | 8 (89%) | 8 (73%) |
| Platelets [E9/l] | 287 (86-538) | 388 (275-597)^a^ | 352 (313-547) | 376 (262-455) |
| Thrombocytopenia [<150 E9/l] | 3 (8%) | 0 (0%) | 0 (0%) | 0 (0%) |
| Thrombocytosis [>360 E9/l] | 21 (53%) | 10 (60%) | 6 (67%) | 8 (73%) |

Subjects characteristics from questionnaire, hospital records and laboratory tests by study group. Results are reported as N (%) and median (range). Only subjects requiring medication listed for hypertension, diabetes, hypercholesterolaemia, and hypothyroidism. Coronary artery disease verified by angiography. ^a^Significant at p<0.05 level compared to the non-GCA group after Bonferroni adjusting for multiple comparisons. ^b^Anaemia: Hb <117g/l for women and Hb <134g/l for men.

Supplemental figure 1. Halo-Doppler ratio and VHRU Intima thickness in relation to corticosteroid treatment.

**
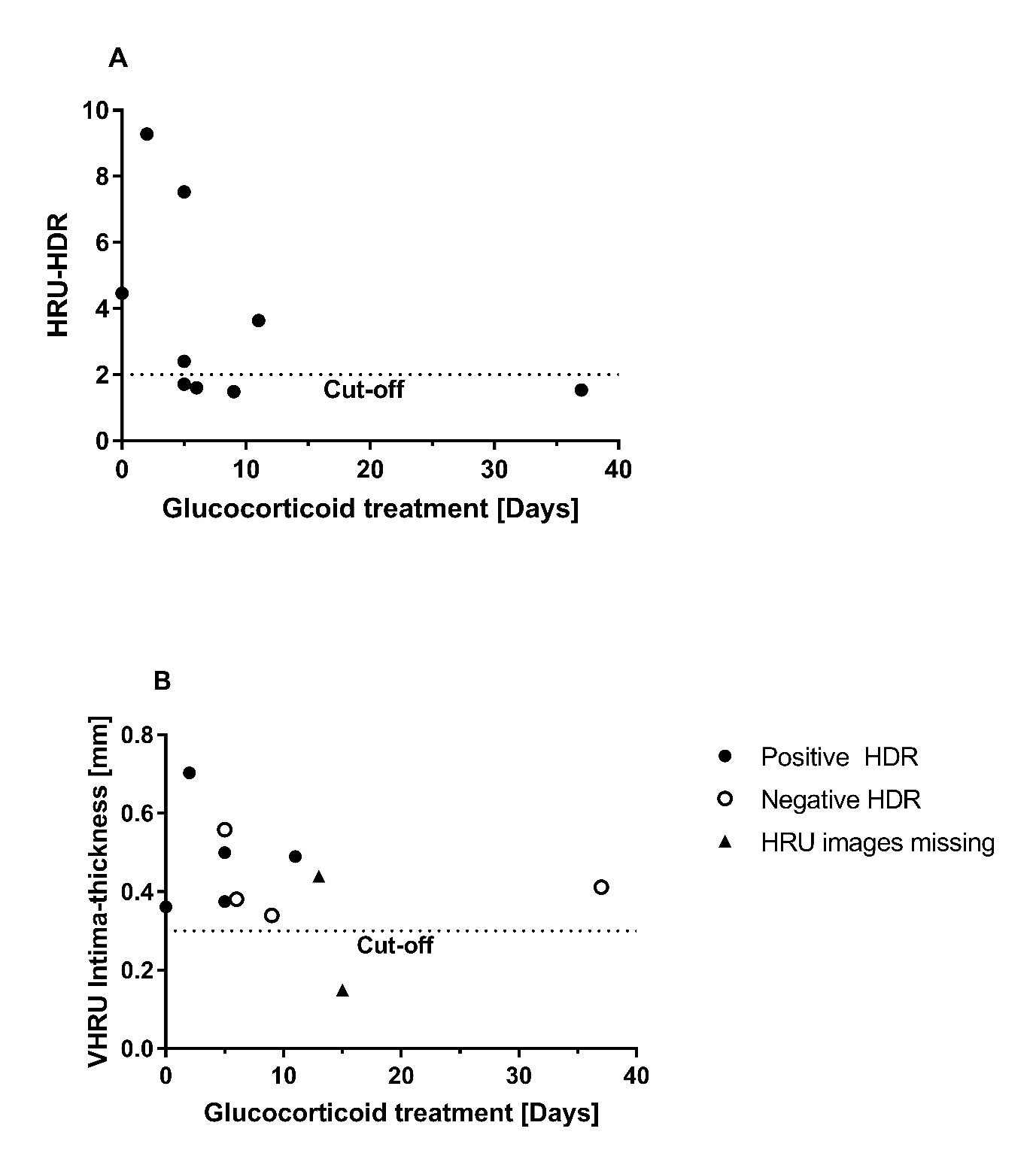
**

Supplemental figure 1. A) Conventional ultrasound derived Halo-Doppler ratio versus corticosteroid-treatment duration prior to imaging in the transmural inflammation (TMI) group. B) VHRU intima thickness versus corticosteroid-treatment duration prior to imaging in TMI group. Note that the intimal thickness exceeds the diagnostic cut-off 0.3mm from 5 days of corticosteroid treatment, whereas the prevalence of the halo-sign diminishes. HDR – Halo-Doppler ratio; HRU – High-resolution ultrasound; VHRU – Very high resolution ultrasound.
